# Composition of the Extracellular Matrix of Lymphatic Novel Threadlike Structures: Is It Keratin?

**DOI:** 10.1155/2013/195631

**Published:** 2013-05-21

**Authors:** Hyub Huh, Byung-Cheon Lee, Sang-Hyun Park, Ji Woong Yoon, Soo Jae Lee, Eun Jung Cho, Seung Zhoo Yoon

**Affiliations:** ^1^Department of Anesthesiology and Pain Medicine, College of Medicine, Korea University, Seoul 136-705, Republic of Korea; ^2^Ki Primo Research Laboratory, Division of Electrical Engineering, KAIST Institute for Information Technology Convergence, Korea Advanced Institute of Science and Technology (KAIST), Daejeon 305-701, Republic of Korea; ^3^Impedance Imaging Research Center & Department of Public Administration, Kyung Hee University, Seoul 130-701, Republic of Korea; ^4^Nanotechnology Research Center, Konkuk University, Chungju 380-701, Republic of Korea

## Abstract

*Background*. The lumen of novel threadlike structures (NTSs) is enclosed by a single layer of endothelial cells surrounded by extracellular matrix (ECM). We hypothesized that collagen may be a component of the ECM associated with lymphatic NTSs. *Methods*. Six female New Zealand white rabbits were anesthetized, and the NTS structures within lymphatic vessels were identified by contrast-enhanced stereomicroscopy or alcian blue staining. Isolated NTS specimens were stained with acridine orange, YOYO-1, and 1,1′-dioctadecyl-3,3,3′,3′-tetramethylindocarbocyanine perchlorate (DiI). The structural and molecular composition of the ECM was investigated using transmission electron microscopy (TEM), electrospray ionization-mass spectrometry, and proteomic analysis. *Results*. The lymph vessel wall was stained red by DiI, and rod-shaped nuclei were stained green by YOYO-1. The area surrounding the NTS was also stained red and contained green rod-shaped nuclei. TEM images showed that the NTS consisted of many ECM fibers and the ECM fibers appeared to be ~100 nm in diameter and had narrowly spaced striated bands. Proteomic analysis of the lymphatic NTS-associated ECM identified 4 proteins: keratin 10, cytokeratin 3, cytokeratin 12, and soluble adenylyl cyclase. *Conclusion*. The TEM study suggested that the lymphatic NTS-associated ECM did not contain collagen. This was confirmed by proteomic analysis, which showed that keratin was the major component of the ECM.

## 1. Introduction

In 2003, Jiang et al. [1] established the existence of intravascular novel threadlike structures (NTSs). Their study was inspired by the Bong-Han theory, which was first introduced by Kim in 1963 [2]. Jiang et al. reinvestigated the Bong-Han theory using modern techniques, and consequently, NTSs were discovered in various organs, including rabbit blood vessels [1], rat lymphatic vessels [3], bovine heart [4], rabbit central nervous system [5], and on the surface of rat abdominal organs (liver, stomach, and hollow viscus) [6, 7]. NTSs have also been called Bonghan ducts or primo vessels by Jiang et al.

Histologically, the structure of the NTS appeared to be a simple bundle of several ductules with characteristic rod-shaped nuclei (10–20 *μ*m long), which were clearly visible by phase contrast microscopy. In cross section, the NTS presents as a tissue containing several small lumens, 6–10 *μ*m in diameter. The ductule lumen is lined by a single layer of endothelial cells surrounded by extracellular matrix (ECM) [8]. However, there have been few studies of the fibrous elements that make up the NTS-associated ECM. 

A previous study, using fluorescent magnetic nanoparticles and in vivo imaging techniques, identified NTSs inside lymphatic vessels [9]. The ECM was shown to consist of loosely packed and randomly distributed collagen fibrils. In a recent report, Jung et al. [10] examined the ECM composition of NTSs at the serosal surface of the large intestine and the liver capsule in rabbits and concluded that the ECM is probably collagen. NTSs associated with the endocardial surface of cattle and the serosal and adventitial surfaces of the rat colon were also reported to contain collagen. In that study, however, the authors did not examine the composition of the ECM in lymphatic NTSs. However, conflicting results were reported by Lee et al. [11]. They examined cross sections of an NTS stained with Masson's trichrome, which is widely used to detect collagen fibers in tissue specimens. Although strong staining was observed in mural cells and the matrix surrounding the lymphatic vessel, the NTS was not stained, suggesting that the lymphatic vessel, but not the NTS, contained collagen.

In this study, we sought to determine if the ECM of lymphatic NTSs is composed of collagen. We identified NTS structures in rabbits by stereomicroscopy using a contrast-enhancing optical method or alcian blue staining. Isolated NTS specimens were stained with acridine orange, YOYO-1, or 1,1′-dioctadecyl-3,3,3′,3′-tetramethylindocarbocyanine perchlorate (DiI) to identify the nuclei, membranes, and ECM. The composition of the ECM was further investigated using TEM, electrospray ionization-mass spectrometry (ESI-MS), and proteomic analysis.

## 2. Materials and Methods

### 2.1. Preparation of Rabbit Tissues

Six female New Zealand white rabbits (~1.8 kg) were housed under conditions of constant temperature and humidity (23°C, 60% relative humidity), with a 12 h light-dark cycle. The rabbits were fasted for 12 h before surgery and then anesthetized by an intraperitoneal injection 1.5 g/kg of urethane or Zoletil. The care and handling of animals were in full compliance with institutional regulations and current international laws and policies (Guide for the Care and Use of Laboratory Animals, National Academy Press, 1996).

The adipose tissue surrounding the inferior vena cava was separated and removed. The lymph vessels inside the caudal vena cava were located, and the NTSs were visualized with a contrast-enhanced optical method using a stereomicroscope (SZX12; Olympus, Tokyo, Japan). Alcian blue solution was added when necessary to distinguish the NTS. The NTS specimens were isolated from the lymphatic vessels and fixed in 4% (wt/vol) paraformaldehyde (PFA) or 10% (vol/vol) neutral-buffered formalin (NBF) for up to 2 days. Specimens were stored at 4°C until further analysis.

### 2.2. Microscopic Identification of Extracellular Matrix

After fixation, the tissues were stained with acridine orange, YOYO-1, or DiI to discriminate between the NTS and lymphatic vessels. The sections were then incubated with antifade reagent (Molecular Probes, Grand Island, NY, USA) and mounted for microscopy. Acridine orange-stained sections were visualized using differential interference contrast microscopy, and sections stained with YOYO-1 and DiI were examined by confocal laser scanning microscopy (LSM 500, Zeiss, Germany).

### 2.3. Transmission Electron Microscopy (TEM)

For examination by TEM, tissues were fixed in 2.5% PFA and 2.5% glutaraldehyde in a neutral 0.1 M phosphate buffer for 1 h. Tissues were postfixed for 1 h in 1% (wt/vol) osmic acid in phosphate-buffered saline (PBS), dehydrated in a graded ethanol series, and embedded in Epon812 (EMS, Fort Washington, PA, USA). Semithin (1 mm) sections were stained with 1% (wt/vol) toluidine blue in 1% borax, observed under a light microscope (Axiophot, Carl Zeiss, Germany) to study the gross morphology, and photographed. Ultrathin sections were cut and mounted on nickel grids and then double stained with uranyl acetate followed by lead citrate. The sections were examined with a JEOL 100 CX-II TEM (Tokyo, Japan).

### 2.4. LC-MS/MS Analysis of Lymphatic NTS

LC-MS/MS analysis of the lymphatic NTS structures was performed as previously described [12]. The isolated lymphatic NTSs were homogenized and sonicated. Samples of 10 *μ*g of homogenate were resolved by 4–12% gradient Tris-glycine PAGE (Invitrogen, Carlsbad, CA, USA). The gel was then sliced into 10 pieces from the bottom to the top of the gel, and in-gel digestion was performed by incubation of slices with 10 ng/*μ*L sequencing grade modified trypsin (Promega, Madison, WI, USA) in 50 *μ*L of 50 mM NH_4_HCO_4_ buffer (pH 8.0) at 37°C overnight, as previously described [13]. The tryptic peptides were then loaded onto a fused silica microcapillary C18 column (75 *μ*m × 10 cm). LC separation was conducted under a linear gradient as follows: 0 min, 3% B; 5 min, 3% B; 75 min, 40% B; 80 min, 90% B; 90 min, 90% B; 91 min, 3% B; 110 min, 3% B. The initial solvent was 3% B, and the flow rate was 200 nL/min. Solvent A was 0.1% formic acid in H_2_O, and solvent B was 0.1% formic acid in acetonitrile. The separated peptides were subsequently analyzed using a linear ion trap mass spectrometer (LTQ; Thermo Finnigan, San Jose, CA, USA). The electrospray voltage was set at 2.0 kV and the threshold for switching from MS to MS/MS was 250. All spectra were acquired in data-dependent mode and each MS scan was followed by MS/MS scans of the 3 main peaks from each MS scan.

All MS/MS spectra were searched against the rabbit whole protein database using the SEQUEST algorithm. Dynamic modifications were permitted for oxidized methionine (+16 Da) and carboxyamidomethylated cysteine (+57 Da). The SEQUEST criteria for identifying peptides included *Xcorr* values of at least 1.8, 2.3, and 3.5 for the +1, +2, and +3 charged peptides, respectively.

## 3. Results

**Figure 1:**
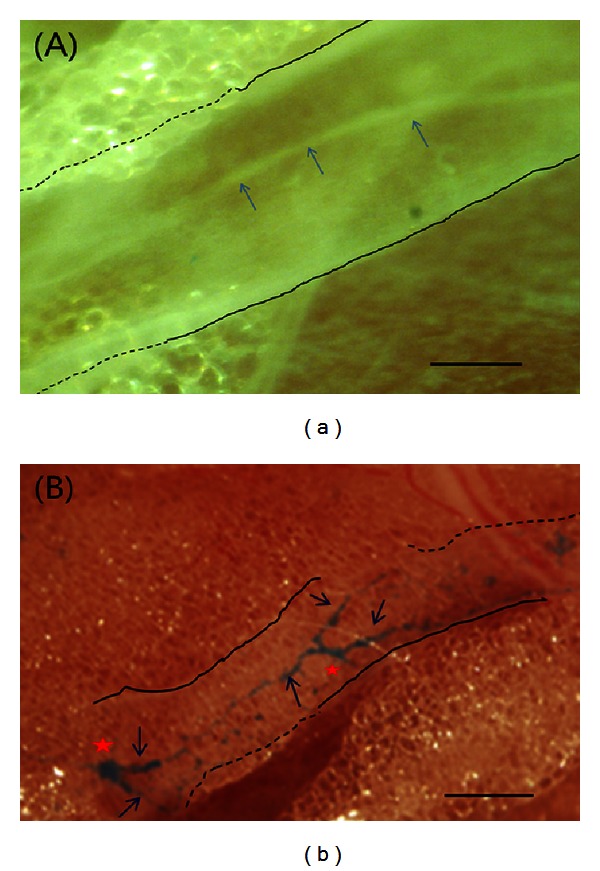
Stereoscopic images of a novel threadlike structure (NTS) in a rabbit lymphatic vessel: (a) contrast-enhanced optical image, (b) alcian blue-stained image. (a) The outermost wall of the lymphatic vessel is indicated by solid black lines (exposed portion) and dotted lines (embedded in fat). The contrast-enhanced NTS (arrows) is freely movable within the lymphatic vessel of the anesthetized rabbit (see movie in Supplementary Material available online at http://dx.doi.org/10.1155/2013/195631). (b) NTS visualized after injection of alcian blue into the rabbit lymphatic vessel. The NTS (arrows) is floating inside the lymphatic vessel, indicated by black lines as in (a). Note the 2 branch points (red asterisks) within the lymphatic vessel. Scale bars in (a) and (b) are 100 *μ*m and 200 *μ*m, respectively.

Lymphatic vessels containing NTSs were identified by stereomicroscopy using a contrast-enhanced optical method or alcian blue staining. The contrast-enhanced optical method was used for 2 rabbits, and the NTS could be observed moving up and down in rhythm with the breathing pattern (Figure 1(a), movie available in Supplementary Materials). For the remaining 4 rabbits, alcian blue dye was injected into the lymphatic vessels around the caudal vena cava. In Figure 1(b), the blue-colored NTS can be seen within the lymph vessel in 2 segments (Figure 1(b)).

**Figure 2:**
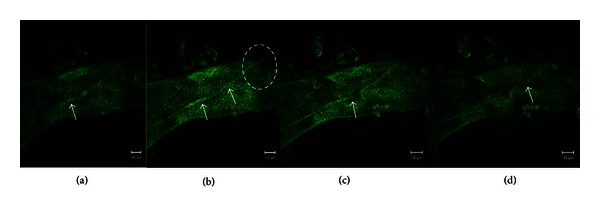
Confocal laser scanning microscopic images of a novel threadlike structure (NTS). Images show serial optical sections (panels (a–d); 1 *μ*m thick) of an NTS-containing lymphatic vessel stained with acridine orange. The arrows indicate the NTS within the lymphatic vessel. Panel (b) shows the distinct protrusion of the NTS from the lymphatic vessel (dotted oval). Scale bar in all panels is 100 *μ*m.

**Figure 3:**
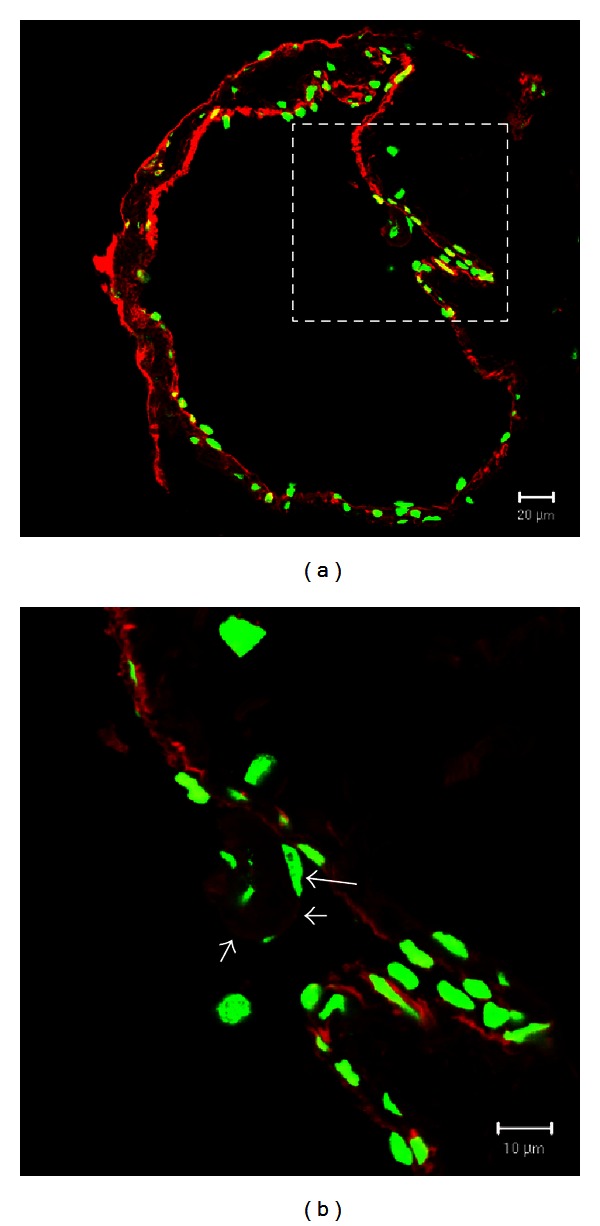
Confocal laser scanning microscopic images of cross sections of a novel threadlike structure (NTS) within a rabbit lymphatic vessel. (a) The lymphatic vessel wall is stained red (DiI), and the rod-shaped nuclei (YOYO-1; green) are visible along the vessel wall. The dotted square is magnified in (b). (b) The long arrow indicates the nucleus in the outermost membrane, and the 2 short arrows and the asterisk indicate the NTS-associated outermost membrane and nucleus, respectively. Scale bars in (a) and (b) are 20 *μ*m and 10 *μ*m, respectively.

Sections of the lymphatic vessels containing the NTS were stained with acridine orange, YOYO-1, and DiI dyes to visualize the nuclei and membranes. Sections stained with acridine orange were examined by confocal laser scanning microscopy (Figure 2). The specimen was optically sectioned into 4 serial images from the top of the lymph vessel. The images of NTS inside the lymph vessels were gradually clarified toward the bottom.  Figure 3 shows a cross section of the specimen stained with YOYO-1 and DiI fluorescent dyes. The lymph vessel wall is stained red by DiI, and the green rod-shaped nuclei (YOYO-1) are visible along the lymph vessel wall (Figure 3(a)). At higher magnification (Figure 3(b)), we observed that the tissue surrounding the NTS is also stained red and contains green rod-shaped nuclei.

**Figure 4:**
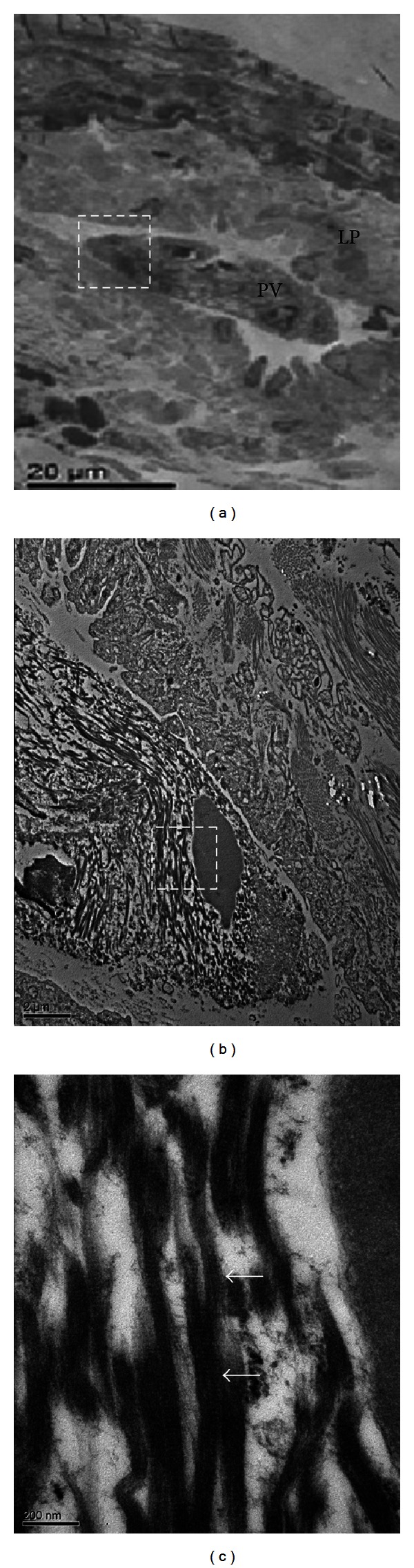
Transmission electron microscopic image of cross sections of a novel threadlike structure (NTS) within a rabbit lymphatic vessel. (a) A low-magnification image of an NTS contained within the lymphatic vessel. The dotted square is magnified in (b). (b) The NTS consists of many extracellular fibers (asterisks). Note the nucleus-like highly electron-dense structure (arrow) in the sinus. This structure corresponds to the asterisk in Figure 3(b). (c) High-power magnification of the dotted square in (b), which shows distinctive fibers (~100 nm in diameter) with narrowly spaced striations (arrows). Scale bars in (a), (b), and (c) are 20 *μ*m, 2 *μ*m, and 200 nm, respectively.

To examine the structure of the NTS-associated ECM, we performed TEM imaging on the same specimen. The TEM images show an NTS composed of many ECM fibers and a highly electron-dense structure in the sinus (Figure 4). This structure corresponds to the image shown in Figure 3(b). Under high magnification, the ECM fibers appear to be ~100 nm in diameter and contain narrowly spaced striated bands (Figure 4(c)).

We performed proteomic analysis of a lymphatic NTS homogenate to identify the protein components of the ECM. We identified 4 proteins: keratin 10 (GI number 87045985), cytokeratin 3 (GI number 75059267), cytokeratin 12 (GI number 3183048), and soluble adenylyl cyclase (GI number 126723185). These proteins were identified using high stringency cutoff *Xcorr* values and consensus scores as criteria for the identification of peptides and proteins, respectively. Only proteins with a consensus score greater than 10.1 were selected. The small number of proteins found may have been due to the very small amount of protein (total 1.682 *μ*g) available for analysis.

## 4. Discussion

We hypothesized that collagen may be one component of the lymphatic NTS ECM. Unexpectedly, our results suggest that this is not the case.

The main difficulty associated with identifying the NTS in vivo and in situ is discriminating between the threadlike structure and fibrin strings. With acridine orange staining, however, fluorescence microscopy can be used to identify nuclei and thus distinguish the NTS from the fibrin strings. The nuclei of the NTS tissue are rod shaped, 10–20 *μ*m long, and are aligned in a broken line. In contrast, the cell nuclei associated with the fibrin strings are globular and randomly scattered; these are actually the nuclei of fibrin-associated white blood cells. This method of positively identifying the NTS is a major breakthrough that allowed us to identify and firmly establish the existence of the NTS inside blood vessels.

The fine network structure of the NTS was not obvious with the alcian blue or acridine orange stains but was clearly revealed by DiI, a highly lipid-soluble cell membrane dye that has been used extensively as a retrograde and anterograde tracing agent in nerve tissue [14]. The tracing property of DiI occurs by a process of lateral diffusion [15]. The DiI labeling of fine networks and terminal arborizations of NTS observed in this study were likely to be due to the mode of action of DiI and not by lateral diffusion in the proximal region of the NTS, because the DiI fluorescence was not continuous and restrained within the sinuses or in the spaces surrounding the sinuses. Given its minimal cytotoxicity and long-term stability restrained to within sinuses or surrounding spaces in animals, DiI appears to be a promising dye for the analysis of the fine morphology and functions of NTSs [16]. 

The main limitation of DiI is that it cannot stain DNA. The alignment of rod-shaped nuclei along the major axis of the NTS is one of the characteristics that can discriminate the NTS from other similar-looking tissues or artifacts. Therefore, we simultaneously stained the specimens with the DNA-specific dye YOYO-1. As shown in Figure 3, the rod-shaped nuclei are visible along the lymph vessel wall, and rod-shaped nuclei in the NTS were observed at higher magnification (Figure 3(b)). These findings provided confidence that the specimen analyzed by TEM was indeed the ECM of a lymphatic NTS.

Transmission electron microscopy (TEM) is a technique whereby a beam of electrons is transmitted through an ultrathin specimen, interacting with the specimen as it passes through. TEM is capable of imaging at a significantly higher resolution than light microscopy, owing to the small de Broglie wavelength of electrons. This enables examination of detail as small as a single column of atoms, which is tens of thousands of times smaller than the smallest object resolvable by light microscopy. Images of collagen fibrils obtained by TEM have shown that collagen monomers align parallel to the main axis of the fibril in a staggered manner. In negatively stained TEM images of collagen fibrils, the banding pattern is ascribed to stain penetration and deposition into vacant spaces between monomers, and thus, the periodic dark bands along these fibrils are commonly referred to as “gap” regions or zones. The banding periodicity of alternating dark and light bands of collagen have been shown to be ~67 nm and ~70 nm, respectively [17]. However, the ECM fibers shown here (Figure 4(c)) show a pattern of narrowly spaced striations that are distinct from the pattern observed in TEM images of known collagen fibrils. 

What, then, is the main component of lymphatic NTS ECM? Our results suggest that it may be keratin. Although we identified only 4 proteins, due to the small amount of protein available, 3 of the proteins were keratins (Krts): Krt10, cytokeratin 3, and cytokeratin 12. Our results are consistent with a recent proteomic study [12] that identified 3 keratins in the NTS tissues: Krt3, Krt10, and Krt12. Keratins are cytoskeletal proteins important for the integrity and stability of epithelial cells and tissues [18]. Krt10, which is the most abundant of the 3 keratins detected in NTS tissue, pairs with Krt1, a type II keratin, to form dense bundles that are features of suprabasal epidermal keratinocytes in humans. These Krt10/Krt1 pairs provide mechanical integrity to the epidermis. Krt10 is normally expressed in postmitotic keratinocytes, where it inhibits keratinocyte proliferation and cell cycle progression and reduces skin tumorigenesis [19]. In addition, Krt10 is a duct-specific marker in normal eccrine sweat glands [20]. In a subsequent study, Kim et al. [21] also demonstrated Krt10 expression in NTS tissues on the surface of abdominal organs in the rat. Krt10 was visualized in patchy spots around single cells or in follicle-like structures containing groups of cells but was not detected on the external membranes of NTS nodes. Krt10 was also identified in blood and lymphatic vessels, but its distribution was diffused. 

## 5. Conclusion

The results of our TEM study suggested that the lymphatic NTS-associated ECM might not contain collagen, as was suggested by previous studies using trichrome staining. Instead, our proteomic analysis suggests that keratin is present. Although the proteomic study was limited by the small amount of protein analyzed (1.682 *μ*g), it is very meaningful to share the same implication with TEM images, however, to further functional research of lymphatic NTS because this is the first try to identify the proteins in lymphatic NTS.

## Supplementary Material

Supplementary Material: The contrast-enhanced optical method video of a novel threadlike structure (NTS) in a rabbit lymphatic vessel. The contrast-enhanced NTS is freely movable within the lymphatic vessel of the anesthetized rabbit.
